# Venous thromboembolism in anti-neutrophil cytoplasmic antibody-associated vasculitis: an underlying prothrombotic condition?

**DOI:** 10.1093/rap/rkaa056

**Published:** 2020-10-16

**Authors:** Aleksandra Antovic, Einar Svensson, Björn Lövström, Vera Bäckström Illescas, Annica Nordin, Ola Börjesson, Laurent Arnaud, Annette Bruchfeld, Iva Gunnarsson

**Affiliations:** r1 Department of Medicine, Division of Rheumatology, Karolinska Institutet; r2 Unit of Rheumatology, Karolinska University Hospital, Stockholm, Sweden; r3 Department of Rheumatology, Hôpitaux Universitaires de Strasbourg, Centre National de Références des Maladies Systémiques et Autoimmunes Rares Est Sud-Ouest (RESO), Université de Strasbourg, Strasbourg, France; r4 Department of Renal Medicine, Karolinska University Hospital and CLINTEC Karolinska Institutet, Stockholm, Sweden

**Keywords:** venous thromboembolism, ANCA-associated vasculitis, global haemostatic assays

## Abstract

**Objectives:**

We investigated the incidence and potential underlying risk factors of venous thromboembolism (VTE) in patients with AAV. We assessed haemostatic disturbances and factors that might contribute to the risk of development of VTE.

**Methods:**

ANCA-positive AAV patients (*n* = 187) were included. Previously identified risk factors for VTE and current medication were retrieved from the medical records. We assessed haemostasis using different methods [endogenous thrombin potential (ETP), overall haemostatic potential (OHP), overall coagulation potential (OCP) and overall fibrinolysis potential (OFP)] in patients with active AAV (*n* = 19), inactive AAV (*n* = 15) and healthy controls (*n* = 15).

**Results:**

Twenty-eight VTEs occurred in 24 patients over a total follow-up time of 1020 person-years. A majority of VTEs occurred within the first year after diagnosis. Old age (*P* < 0.01), ongoing prednisolone treatment and recent rituximab administration were more common in the VTE group (*P* < 0.05 for all). ETP and OHP were significantly increased and OFP significantly decreased in plasma from active compared with inactive AAV patients (*P* < 0.05, *P* < 0.01 and *P* < 0.05, respectively) and healthy controls (*P* < 0.001). We could not confirm previously reported risk factors for VTE development.

**Conclusion:**

A high prevalence of VTE in AAV patients was seen within the first year after diagnosis, suggesting that disease activity contributes to development of VTE. Old age and concurrent treatment should also be taken into account when estimating VTE risk. The results also indicate disturbances in the haemostatic balance towards pro-thrombotic conditions in AAV patients, where ETP and OHP might be useful markers for identifying patients at high risk.

**Key messages**

- Venous thromboembolism is a common complication in AAV and occurs predominantly in early disease.
- Old age and concurrent treatment should be taken into account when estimating the risk for venous thromboembolism.
- Disturbances in the haemostatic balance towards pro-thrombotic conditions may influence the risk for venous thromboembolism.

## Introduction

AAV covers three different clinical entities: granulomatosis with polyangiitis (GPA), microscopic polyangiitis (MPA) and eosinophilic granulomatosis with polyangiitis (EGPA). They are a group of systemic diseases characterized by necrotizing vasculitis and granulomatous inflammation, mainly affecting small- to medium-sized vessels, and are differentiated by subtle differences in the clinical phenotype [1, 2]. An increased incidence of venous thromboembolic events (VTE) has been recognized among AAV patients compared with the general population [3–7], but also in patients with other chronic inflammatory diseases, such as SLE, myositis and RA [8, 9]. Merkel *et al*. [4] were the first to show that a majority of the VTEs occurred in patients with active or recently active vasculitis. The relationship between disease activity and the occurrence of VTE has later been confirmed in subsequent retrospective cohort studies [5–7]. 

Data regarding the role of classical risk factors for VTE in patients with AAVs are, to a large extent, lacking. Several studies have failed to show a connection between the prevalence of acquired or genetic risk factors for VTE and the increased incidence of VTE in AAV patients [3, 6, 10]. Furthermore, the clinical and serological features of AAV patients with VTE are not fully known, and the role of ANCA specificity as a risk factor for VTE has previously given conflicting data. Some previous studies have shown an increased frequency in PR3-positive individuals and development of VTE [3, 11] whereas others have found a lower frequency among GPA and with PR3 [6], or no difference in ANCA specificity at all [5, 12]. Furthermore, an association between VTE and subsequent development of malignancies has also been suggested in AAV [13]. 

An increased risk of VTE was recently observed in the early phase of disease in patients from the Rituximab in Antineutrophil Cytoplasmic Antibody (ANCA)-Associated Vasculitis (RAVE) trial, in which patients with active disease were given rituximab treatment. A high incidence of VTE was seen in patients with pulmonary haemorrhage, heart involvement or urinary blood cell casts [11], indicating that a severe disease phenotype might increase the risk of development of VTE.

The present study aimed to determine the incidence of VTE in our cross-sectional cohort of AAV patients and to assess potential risk factors for development of VTE. The connection of disease activity and presence of a hypercoagulable state was further investigated by two global haemostatic methods [overall haemostatic potential (OHP) and endogenous thrombin generation (ETP)] in plasma of AAV patients with active disease and in a group of inactive patients.

## Methods

This was an observational, retrospective cohort study of AAV patients at Karolinska University Hospital from both the rheumatology and nephrology clinics, who have been included in a cross-sectional and longitudinal study (the VASKA study) since 2009. Both prevalent and incident cases were included in the study. Inclusion criteria in the present study were a diagnosis of GPA or MPA, age of ≥18 years and a positive ANCA for MPO or PR3 (ever). In the case of ANCA specificity towards both PR3 and MPO (*n* = 3), the specificity of the higher titre was used in the inferential statistics. All included patients were diagnosed according to the validated EMEA algorithm for epidemiological studies of AAV [14].

The exclusion criteria were ANCA negativity and/or a diagnosis of vasculitis before 2005. A follow-up period of ≥12 months was required for inclusion in the study. Ethical permission had been acquired from the Regional Ethical Review Board in Stockholm, and informed consent was obtained from all study subjects. The study complies with the Declaration of Helsinki.

### Data collection

For the entire cohort, information on sex, autoantibody profiles and diagnosis (GPA or MPA) at the time of AAV diagnosis was retrieved. For the calculation of VTE incidence, all patients were followed from 3 months before the time of diagnosis of vasculitis (a method suggested by Stassen *et al*. [6]) until the end of follow-up, end of medical record information, emigration or death, whichever came first. Patients who developed VTE after the AAV diagnosis were labelled as the VTE group, and patients with no VTE were called the non-VTE group.

Information on previous VTEs, defined as a diagnosis >3 months before the time of AAV diagnosis, was also collected. These VTEs were not included in the calculations of incidence. A VTE was defined as a deep vein thrombosis (DVT), a pulmonary embolism (PE) or both. Only objectively verified VTE diagnoses (for DVT by US or venography, and for PE either by CT pulmonary angiography or pulmonary scintigraphy) were included in the calculations of incidence. In the calculation of VTE incidence, the number of confirmed cases of VTE during AAV was set as the numerator, and the total follow-up time, expressed in 100 person-years, was set as the denominator. Renal involvement was primarily defined as pathological changes on a renal biopsy or by the presence of significant haematuria and/or elevated creatinine values.

Data regarding known risk factors of VTE were retrieved from the medical records at inclusion in the study cohort. This included a diagnosis of diabetes, hypertension, hyperlipidaemia and BMI. A history of smoking status was recorded and defined as current or ever. A history of malignancy, whether previous or current at the time of diagnosis or diagnosed during the follow-up period, was retrieved from the medical records (Table 1).

**Table 1:** Baseline patient and disease characteristics

Parameter	All patients (*n* = 187)	VTE group (*n* = 24)	Non-VTE group (*n* = 163)	*P*-values
Patient and disease characteristics				
Male sex, *n* (%)	89 (48)	12 (50)	**77** (47)	0.8
Age at inclusion or VTE, mean (s.d.)	59.1 (16.8)	69 (11.4)	57.6 (16.9)	0.0012
Disease duration at inclusion or VTE, years, mean (s.d.)	1.23 (1.7)	1.4 (2.2)	1.2 (1.66)	0.14
History of previous VTE, *n* (%)	9 (4.8)	3 (12.5)	6 (3.1)	0.07
GPA, *n* (%)	129 (69)	15 (62.5)	114 (70)	0.46
MPA, *n* (%)	58 (31)	9 (37.5)	49 (30)	0.46
PR3–ANCA, *n* (%)	119 (63.6)	14 (58.3)	105 (64.4)	0.56
MPO–ANCA, *n* (%)	68 (36.4)	10 (41.7)	58 (35.6)	0.56
Renal involvement ever, *n* (%)	126 (67.4)	18 (75)	108 (66.3)	0.39
P-creatinine, μmol/l, mean (range)	144 (41–1032)	151 (57–364)	143 (41–1032)	0.02 (0.33 age adjusted)
Urinary red blood cell casts >3, *n*/powerfield (%)	69/162 ^a^ (42.6)	7/18 ^a^ (38.9)	62/144^a^ (43.1)	0.8
Smoker, ever, *n* (%)	111 (59.4)	16 (66.7)	95 (58.3)	0.43
Smoker, current, *n* (%)	17 (9.1)	1 (4.2)	16 (9.8)	0.7
Co-morbidities				
Hypertension, *n* (%)	89 (47.6)	10 (41.7)	79 (48.5)	0.53
Diabetes, *n* (%)	22 (11.8)	1 (4.2)	21 (12.9)	0.32
BMI, kg/m^2^, mean (s.d.)	26.2 (5.5)	25.4 (3.4)	26.3 (5.8)	0.77
BMI >25 kg/m^2^	98 (52.4)	15 (62.5)	83 (50.9)	0.29
Malignancy known at inclusion/VTE, *n* (%)	10 (5.3)	1 (4.2)	9 (5.5)	1.0
Malignancy during follow-up, *n* (%)	17/186^b^ (9.1)	4/24 (16.7)	13/162^b^ (8.0)	0.24
Follow-up				
Total follow-up time since inclusion or VTE, years	1020	148	872	
Follow-up time, years, mean (range)	5.3 (0–12)	6 (1.5–10.1)	5.3 (0–12.0)	0.08

Data are on the total cohort and the two patient subgroups (with or without VTE). Data on VTE patients were obtained at the time of VTE; data on non-VTE patients were obtained at inclusion in the study cohort. *P*-values were calculated by comparing the two patient subgroups. Values within parentheses are percentages unless stated otherwise. ^a^Data missing in subpopulations. ^b^No follow-up data available. P-values in italic remain significant after Bonferroni correction for multiple testing. GPA: granulomatosis with polyangitis; MPA: microscopic polyangitis; VTE: venous thromboembolism.

Data on current medication (prednisolone usage and dose, DMARD, CYC, biological treatment with rituximab, SAS, warfarin, statins and treatment for hypertension) were recorded at the time point of inclusion in the study in the non-VTE group, or at the onset of VTE in the VTE subgroup (Table 2). In patients used for analysis of haemostasis variables, we recorded the current disease activity using the Birmingham Vasculitis Activity Score (BVAS) [15].

**Table 2:** Treatment at the time of inclusion or at venous thromboembolism development

Parameter	All patients (*n* = 187)	VTE group (*n* = 24)	Non-VTE group (*n* = 163)	*P*-value
Treatment at inclusion or onset of VTE				
Prednisolone, *n* (%)	148 (79.1)	23 (95.8)	125 (76.7)	0.031
Prednisolone dose, mg/day, median (range)	10 (0–80)	15 (0–60)	8.25 (0–80)	0.3
DMARD, *n* (%)	91 (48.7)	12 (50)	79 (48.5)	0.89
MTX, *n* (%)	32 (17.1)	3 (12.5)	29 (17.8)	0.77
AZA, *n* (%)	35 (18.7)	4 (16.7)	31 (18.6)	1.0
MMF, *n* (%)	24 (12.8)	5 (20.8)	19 (11.7)	0.2
CYC, *n* (%)	50 (26.7)	9 (3.8)	41 (25.2)	0.2
Rituximab (within 3 months), *n* (%)	6 (3.2)	4 (16.7)	2 (1.2)	0.003
Warfarin, *n* (%)	6 (3.2)	0 0	6 (3.7)	1.0
ASA, *n* (%)	24 (12.8)	4 (16.7)	20 (12.3)	0.52
Statins, *n* (%)	32 (17.1)	3 (20.8)	29 (17.8)	0.77
RAS blockade, any (ACEi, ARB or both), *n* (%)	75 (40.1)	7 (29.2)	68 (41.7)	0.27
ACEi, *n* (%)	50 (26.7)	4 (16.7)	46 (28.2)	0.32
ARB, *n* (%)	30 (16.0)	4 (16.7)	26 (16)	1.0
Diuretics, *n* (%)	41 (21.9)	7 (29.2)	34 (20.9)	0.43
β-Blockers, *n* (%)	52 (27.8)	4 (16.7)	48 (29.4)	0.23
Calcium blockers, *n* (%)	34 (18.2)	1 (4.2)	33 (20.2)	0.08

Data are on the total cohort and the two patient subgroups (with or without VTE). Data on VTE patients were obtained at the time of VTE. *P*-values were calculated by comparing the two patient subgroups. Figures within parentheses are percentages unless stated otherwise. None remain significant after Bonferroni correction for multiple testing. ACEi: angiotensin-converting enzyme inhibitor; ARB: angiotensin II receptor blocker; RAS blockade: renin–angiotensin system blockade; VTE: venous thromboembolism.

### Investigation of laboratory variables

Plasma creatinine was analysed according to clinical routine at the Clinical Chemistry Department at the Karolinska University Hospital, with values expressed in micromoles per litre. The occurrence of red cell casts in urine sediment analysis was analysed, and levels >3 (number/powerfield) were regarded as increased. We used creatinine values and urine sediment findings from the time point of inclusion in the VASKA study in the non-VTE group and data from the VTE time point in the VTE group.

Detection of ANCA was performed by ELISA (BioPlex 2200 Vasculitis Reagent Pack, Clinical Diagnostics, Bio-Rad) or capture ELISA methods (Wieslab Cap PR3 ANCA kit Euro Diagnostica), at any time point. The analyses were done at the Department of Clinical Immunology according to clinical routine.

### Investigation of global haemostasis

We investigated two global haemostatic methods, endogenous thrombin potential (ETP) and overall haemostatic potential [OHP, including overall coagulation potential (OCP) and overall fibrinolysis potential (OFP)], using citrate plasma samples of patients with either active disease (*n* = 19; BVAS ≥1) or in the inactive disease phase (*n* = 15; BVAS = 0). Among the inactive patients, six belonged to the initial study cohort, and available samples from another nine patients were used for the analysis. As controls, we used plasma samples from 15 healthy individuals without a previous history of cardiovascular disease, VTE and malignancy. Among the controls, there were eight males and seven females; mean age 66 (53–80) years. None of the investigated subjects was treated with CSs or anticoagulant drugs at the time point of blood sampling. For information on demographics, renal function and treatment in the AAV subcohorts, see Table 3.

**Table 3:** Clinical characteristics and levels of haemostatic variables in patients with active and inactive AAV

Characteristic	Active AAV (*n* = 19)	Inactive AAV (*n* = 15)	*P*-value
Age	60.5 (15.5)	59.1 (15.3)	ns
Sex, male/female	10/9	6/9	
GPA/MPA	12 GPA/7 MPA	15 GPA/0 MPA	
BVAS	14 (9.3)	0	
Plasma creatinine, mg/day	131 (89.4)	89 (24.7)	0.045
Prednisolone, mg/day, mean (range)	32.9 (0–80)	6.2 (0–20)	0.011
Methylprednisolone	2	0	
DMARD, any	6	12	0.15
MTX	4 (21.1)	5 (33.3)	0.46
AZA	0 (0)	4 (26.7)	0.03
MMF	2 (10.5)	3 (20)	0.63
ASA	0 (0)	2 (13.3)	0.19
Warfarin, ongoing	0 (0)	0 (0)	1
Antihypertensive treatment (any)	9 (47.4)	8 (53.3)	1.0
ACEi	1 (5.3)	4 (26.7)	0.15
ARB	3 (15.8)	5 (33.3)	0.42
β-Blockers	5 (26.3)	7 (46.7)	0.29
Diuretics	2 (10.5)	1 (6.7)	1.0
Statins	3 (15.8)	2 (13.3)	1.0
Malignancy, ever	1^a^	0	
Haemostasis markers			
OCP (Abs-sum)	27.9 (1.5)	24.7 (1.5)	0.06
OHP (Abs-sum)	23.2 (1.3)	17.4 (1.4)	<0.01
OFP (%)	16.7 (2.0)	30.2 (2.3)	**<0.0001**
ETP (mA)	104.9 (2.5)	93.6 (2.7)	0.019

Variables are presented as the mean (s.e.) unless stated otherwise. *P*-values in bold remain significant after Bonferroni correction for multiple testing. ^a^Previous breast cancer. Abs-sum: Sum of absorbance values; ACEi: angotensin-converting enzyme inhibitor; ARB: angiotensin receptor II blocker; BVAS: Birmingham Vasculitis Activity Score; ETP: endogenous thrombin potential; GPA: granulomatosis with polyangitis; MPA: microscopic polyangitis; ns: not significant; OCP: overall coagulation potential; OFP: overall fibrinolytic potential; OHP: overall haemostatic potential.

### Determination of OHP in plasma

According the method described by He *et al*. [16], the OHP assay is based on the construction of fibrin aggregation curves using citrated plasma, into which 0.04 U/ml thrombin (Sigma-Aldrich, USA), 17 mmol/l CaCl_2_ and 300 ng/ml tissue plasminogen activator (t-PA; Boehringer Ingelheim, Germany) were added. Absorbance (Abs) at 405 nm was measured every minute for 40 min. The area under the curve was calculated by summation of the Abs values (Abs-sum). Two additional parameters were also analysed: overall coagulation potential (OCP), determined as the area under the fibrin aggregation curve obtained from citrated plasma, into which 0.04 U/ml thrombin and 17 mmol/l CaCl_2_ were added, and overall fibrinolysis potential (OFP), calculated as the difference between the two areas by: OFP (%) = [(OCP − OHP)/OCP] × 100.

### Determination of ETP in plasma

The ETP assay (for research use only; Siemens Healthcare Diagnostics Products, Marburg, Germany) was performed on a BCS XP System according to the manufacturer's instructions. The ETP value was expressed as miliabsorbance (mA). Thrombin generation curves were visualized and analysed using the curve evaluation software (for research use only; Siemens Healthcare Diagnostics Products).

### Blood sampling

Peripheral venous blood was collected into Vacutainer tubes (Becton Dickinson) containing trisodium citrate (0.129 mol/l, pH 7.4; one part trisodium citrate and nine parts blood). Plasma was obtained within 60 min of sampling by centrifugation at 2000***g*** for 20 min at room temperature, then divided into aliquots and stored frozen at −70°C.

### Statistics

Descriptive statistics were used for presentation of patient characteristics. For continuous variables, means and standard deviations or medians with ranges were used, whereas categorical variables were presented as percentages. Differences between patient categories were analysed using the Mann–Whitney *U*-test for continuous variables, or with Fisher’s exact test or Pearson χ^2^ test (for larger samples) for categorical variables. Bonferroni correction was performed for multiple testing adjustment. A two-sided *P*-value <0.05 was considered statistically significant. Statistical analyses were made using the statistical software program JMP, v.12.1.0 (SAS Institute Inc., Cary, NC, USA).

## Results

A total study group of 187 patients was included, in which 24 patients suffered at least one VTE (VTE group). All data on the VTE patients were retrieved at the time point of the first VTE occurring after AAV diagnosis; for the non-VTE patients, we recorded data from the time of inclusion in the VASKA study. The mean (s.d.) disease duration was 1.23 (1.73) years [1.38 (2.18) years for the VTE group and 1.21 (1.65) years for the non-VTE group, *P* = 0.14].

In the total patient group, 89 patients (48%) were male, the mean age was 59.1 (16.8) years, 129 (69%) of the patients had a diagnosis of GPA, and 121 (64.7%) of the patients were PR3-ANCA-positive (ever). There was no significant difference in sex, diagnosis (GPA *vs* MPA), or ANCA specificity between the VTE group and the non-VTE group [not significant (ns)]. Despite having similar disease duration, the VTE patients were older compared with non-VTE individuals (*P* = 0.001). The baseline characteristics of the patients are shown in Table 1.

A history of previous VTE was more commonly seen in the VTE group compared with non-VTE patients (three in the VTE group, six in the non-VTE group), but not statistically significant (*P* = 0.07). There was no difference regarding renal involvement (ever), but the VTE group had higher plasma creatinine concentrations (mean 151 *vs* 143 μmol/l) compared with non-VTE patients (*P* = 0.02), although this difference did not remain significant (*P* = 0.33) after adjustment for age. Occurrence of significant urinary red blood cell casts did not differ between the groups (ns). Smoking habits, either previous or current, occurrence of diabetes or a diagnosis of hypertension did not differ between the two groups (ns). There was no difference in BMI between the groups (ns; Table 1).

### Malignancies and VTE risk

In the VTE group, one patient (4.2%) had a previous diagnosis of cancer (prostate 24 years prior) before the occurrence of VTE, and four patients (16.7%) were diagnosed with malignancy 4–9 years after VTE diagnosis. Nine patients (5.5%) in the non-VTE group had a history of cancer at the inclusion time point, and 13 patients (8%) developed subsequent carcinoma. There was no significant difference between the groups in the prevalence of cancer either at baseline or follow-up (ns; Table 1).

### Incidence of VTE during follow-up

Twenty-eight VTEs occurred in 24 patients after AAV diagnosis over a total follow-up time of 1020 person-years, with a total incidence rate of 2.74 cases of VTE per 100 person-years. Of the 28 VTEs, 15 were PEs, 9 were DVTs and 4 were cases of simultaneous DVT and PE. The incidence rate of PE in the cohort was 1.86 cases per 100 person-years, and the incidence rate of DVT was 1.27 cases per 100 person-years (when including the cases of simultaneous thromboses in both the calculations). Two patients suffered one recurrent VTE each, and one patient had two recurrent VTEs. The follow-up times in the two groups are presented in Table 1.

**Fig. 1:**
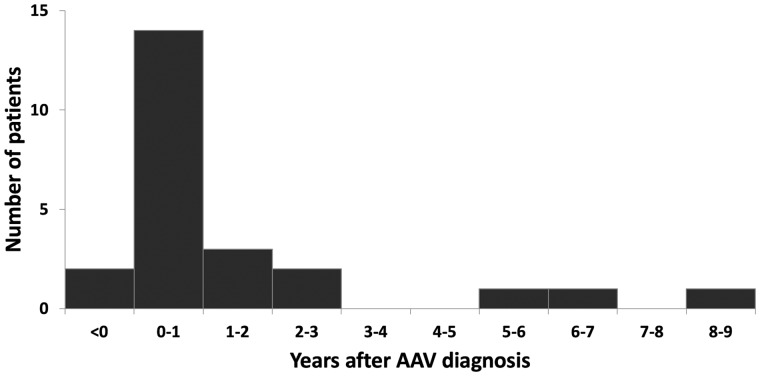
The relationship between AAV duration and occurrence of first venous thromboembolism after diagnosis (*n* = 24) VTE: venous thromboembolism.

The incidence rate of first-time VTE since AAV diagnosis was 2.64 cases per 100 person-years (24 VTEs in 909 person-years of follow-up). Of the first-time VTEs, 13 were PEs, 7 were DVTs and 4 were simultaneous cases. The incidence rate of first-time PE was 1.87 cases per 100 person-years, and the incidence rate of first-time DVT was 1.21 cases per 100 person-years. The median time between AAV diagnosis and first VTE was 3.9 months (−0.9 to 99.6 months). A majority of VTEs occurred in close temporal proximity to the AAV diagnosis, with more than half of the patients having a VTE within the first year after the AAV diagnosis (Fig. 1).

### Treatment

Among all 187 patients, 148 (79.1%) were on CS (prednisolone) treatment at the time of inclusion or development of VTE, with doses ranging from 0 to 80 mg/day. Ongoing prednisolone treatment was more common in the VTE patients compared with non-VTE patients (*P* = 0.03; Table 2).

The median prednisolone dose at the time of VTE onset in the VTE group was 15 mg/day. In the non-VTE group, the median prednisolone dose at the time of inclusion in the study was 8.25 mg/day. However, there was no difference between the groups when comparing doses of prednisolone (*P* = 0.3; Table 2).

Ninety-one of the patients were on treatment with DMARDs (MTX, AZA or MMF), with usage not differing between the two groups. There was no difference in ongoing treatment with CYC. A larger proportion of patients with VTE had been treated with rituximab within the last 3 months compared with non-VTE patients (*P* = 0.003; Table 2).

Six of the non-VTE patients but none in the VTE group were treated with warfarin at the inclusion time point. Warfarin was given owing to previous VTE (before AAV diagnosis; *n* = 2), atrial fibrillation (also VTE before AAV diagnosis; *n* = 1), mechanical heart valve (*n* = 2) and thrombi in the left chamber (*n* = 1). Six non-VTE patients and three VTE patients had a history of previous warfarin treatment (*P* = 0.68). In the VTE group, all patients had been treated owing to a VTE occurring before the AAV diagnosis.

There was no difference in usage of ASA between the groups (ns). Treatment with angiotensin-converting enzyme inhibitors or angiotensin receptor blockers was not different between the two groups, nor was the usage of other antihypertensive drugs or statins (Table 2).

### Global haemostatic parameters

**Fig. 2:**
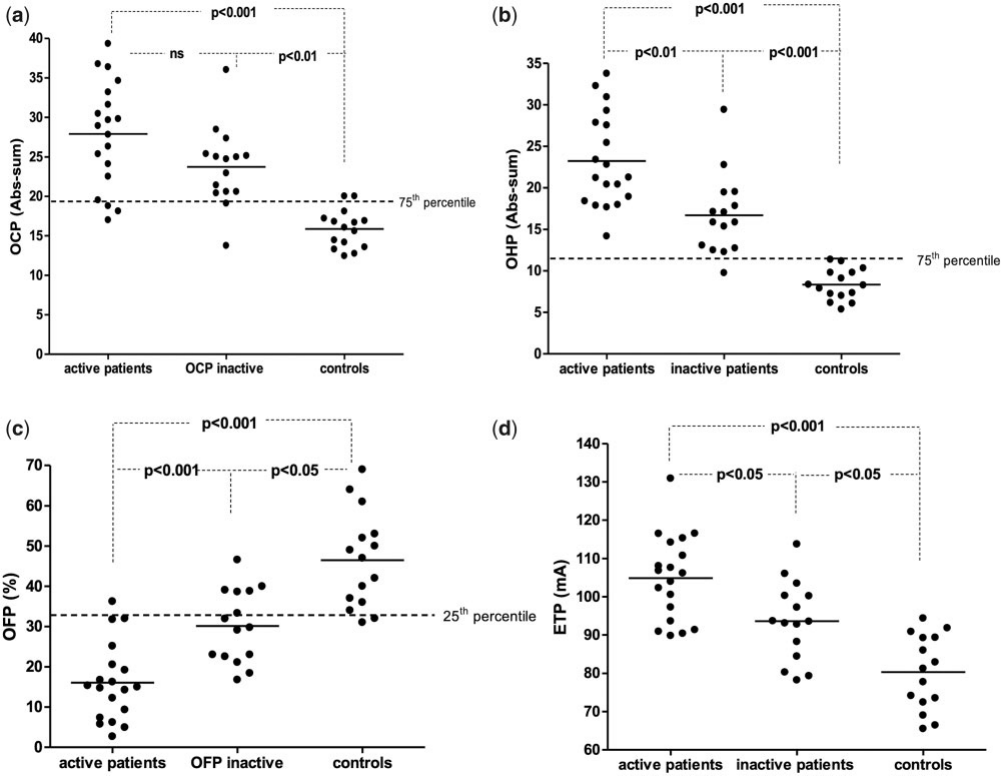
Plasma levels of haemostatic variables in patients with active AAV, patients with inactive AAV and controls (**a**) Overall haemostatic potential (OHP). (**b**) Overall coagulation potential (OCP). (**c**) Overall fibrinolytic potential (OFP). (**d**) Endogenous thrombin potential (ETP).

In the subset of AAV patients investigated for global haemostasis (*n* = 34), there was no significant difference in OCP between active and inactive AAV patients, but both groups of patients had increased OCP compared with the controls (*P* < 0.001 and *P* < 0.01, respectively; Fig. 2a). Patients with active disease had an increased OHP and ETP compared with inactive patients (*P* < 0.01 and *P* < 0.05, respectively) and controls (*P* < 0.001, respectively; Fig. 2b and d). A majority of patients had OCP and OHP levels above 75th percentile of the reference range used in our laboratory (OCP, 12.7–24.0; OHP, 6.5–13.6; Fig. 2a and b).

The OFP was lower in active patients compared with both inactive patients (*P* < 0.001) and controls (*P* < 0.001). Interestingly, inactive patients had lower OFP compared with controls (*P* < 0.05; Fig. 2c). Almost all patients had OFP levels below the 25th percentile of the reference range used in our laboratory (OFP, 31–57%), as demonstrated in Fig. 2c. The levels of the investigated haemostatic variables in the AAV patients are presented in Table 3.

## Discussion

In line with previous studies, we found an overall high incidence of VTE among patients with AAV and a high occurrence in the early and thus active phase of disease. We demonstrated haemostatic disturbances among both active and inactive patients with AAV compared with controls, indicating that AAV *per se* might be associated with an underlying pro-thrombotic state. We found that older age at AAV diagnosis, high CS usage and recent rituximab administration were associated with development of VTE. Not unexpectedly, previous VTE was more common in patients with VTE after AAV diagnosis, but not statistically significant.

In line with a recent study by Kronbichler *et al*. [11], we observed that a majority of the VTEs occurred in close temporal proximity to the diagnosis of AAV. Given the early occurrence of VTE after AAV diagnosis, a relationship between the inflammatory conditions and a pro-thrombotic state must be considered [11, 17–20]. In our study population, VTE was more common in patients with recent (within 3 months) treatment with rituximab compared with other treatment regimens. This contrasts with the findings in the RAVE trial, comparing rituximab and CYC in active AAV patients, where no difference could be seen between the groups [21]. The early VTEs seen in our rituximab-treated patients might be attributed to persistent inflammatory activity rather than effects of the drug itself, and the findings need to be replicated before drawing firm conclusions.

It was demonstrated recently that neutrophil extracellular traps (NETs) and neutrophil-derived microparticles express tissue factor after stimulation by ANCA, which proposes a possible mechanism for activation of coagulation and increased thrombin generation in AAV [12, 22–25]. Another possible contributory mechanism to the prothrombotic state in AAV is impaired fibrinolysis owing to the presence of anti-plasminogen antibodies in AAV patients [26, 27], which could contribute to delayed dissolution of fibrin clots *in vitro*, but this was not investigated in the present study.

In a recent study on a group consisting mainly of GPA patients, the presence of a hypercoagulable state was also found in the remission phase of the disease, owing to the presence of elevated endogenous thrombin generation potential and factor VIII levels when compared with age- and sex-matched healthy controls [28]. We used two global haemostatic assays in the pilot study of AAV patients with active or inactive disease to assess the haemostatic balance. The assessment of single coagulation and fibrinolytic factors and/or inhibitors reveals only a small part of the complex haemostatic process, whereas global haemostatic assays offer an overview of the haemostatic process. Measurement of ETP reflects the total amount and the kinetics of thrombin generated over time in the examined plasma sample, and thereby reflects the thrombotic or bleeding potential of investigated patients [29].

Additionally, the OHP assay provides information concerning the rates of fibrin formation and fibrin degradation, demonstrating the balance between these two opposing haemostatic processes [30]. Interestingly, patients with pre-eclampsia, a hypercoagulable condition, were recently shown to have increased levels of both ETP and OHP compared with healthy pregnant controls. In addition, pre-eclampsia patients with renal involvement had significantly higher ETP values [31].

Our results demonstrated activation of coagulation (by increased levels of OCP, OHP and ETP) and diminished fibrinolysis (OFP) in AAV patients irrespective of disease activity compared with healthy controls. These findings imply an independent effect of the vasculitis itself on the development of a hypercoagulable state [32].

A total VTE incidence of 2.74 cases per 100 person-years in the present study is comparable to those of earlier retrospective studies [3, 6]. As expected, the incidence of VTE in our study was higher than previously shown in the general population (0.1–0.3 cases per 100 person-years) [33, 34], also in comparison with a population with an age range corresponding to that of AAV patients (0.31 cases per 100 person-years) [33]. Interestingly, we observed a higher incidence of PE than of DVT, although the reverse relationship is seen in the general population [34]. One explanation could be the demand for objectively verified VTE diagnoses in our study. The AAV patients suffering PEs might have had concurrent DVTs that were clinically diagnosed but never objectively diagnosed, and thus not included in the incidence calculations.

As in the general population [34, 35], we found older age, and a clear trend of a history of a previous VTE, to be more common in the VTE group. Older age has also been shown previously to be associated with an increased VTE risk among AAV patients [4, 5]. However, we could not confirm an association with male sex and VTE as shown by others [5]. A recent study of AAV patients participating in randomized controlled trials conducted by the European Vasculitis Society demonstrated an association with subsequent development of malignancy in VTE patients [13]. However, our study could not confirm such a relationship, possibly owing to sample size.

In the present study and in line with previous reports [5, 12], no difference in VTE occurrence with respect to ANCA specificity was found. Nevertheless, a review from 2012 recommended that primary thrombo-prophylaxis should be taken into consideration in PR3–ANCA-positive patients with other concurrent classical VTE risk factors [22]. When considering our results, this recommendation should probably also include MPO–ANCA-positive patients.

The high incidence of development of VTE in AAV patients in the early disease phase indicates that a subgroup of patients might benefit from thrombo-prophylactic treatment. However*,* the side effects of anticoagulation therapy have been described in case reports of AAV patients with VTEs, causing severe nasal and gastrointestinal bleeding [36] and pulmonary haemorrhage [37]. Identification of patient subsets with a high-risk profile is thus of major importance to avoid the development of VTE but also to minimize bleeding complications in non-risk groups.

The major strength of this cohort study is the large number of patients from both rheumatological and nephrological clinics, thus covering all disease phenotypes and grades of severity. By requiring the VTEs to be verified objectively, the certainty of the outcomes was improved, and the risk of including misdiagnosed VTEs and thereby overestimating the incidence was minimized*.* This study also has some limitations. Given that the study relied, in part, on data retrieved from Karolinska University Hospital medical records, VTEs diagnosed at other locations might have been missed. Unfortunately, data on disease activity at the time of VTE could not be assessed in our retrospective cohort*.* Further studies are required to determine whether newly diagnosed, active and/or elderly AAV patients would benefit from primary thrombo-prophylactic therapy during the induction treatment phase to lower the incidence of VTE.
